# Sturge-Weber syndrome type III in pediatrics: a case report

**DOI:** 10.3389/fradi.2026.1767963

**Published:** 2026-03-12

**Authors:** Huihui Lin, Xiaoyu Wang, Jing Fan

**Affiliations:** Department of Radiology, Anhui Provincial Children’s Hospital, Hefei, China

**Keywords:** computed tomography, leptomeningeal capillary malformation, magneticresonance imaging, pediatrics, Sturge-Weber syndrome type III

## Abstract

Sturge-Weber syndrome (SWS) type III is a rare neurocutaneous disorder characterized by isolated neurological involvement without cutaneous or ocular manifestations. This case report describes the clinical, laboratory, and imaging findings of a 5-year-old male patient with SWS type III who presented with recurrent headaches and convulsions for 1 year. No facial hemangioma was noted on physical examination. Laboratory tests revealed mild hyperammonemia (29.4 μmol/L; reference range: 10–27 μmol/L) and low pyruvate (18 μmol/L; reference range: 20–100 μmol/L). Video electroencephalogram showed diffuse slow wave elevation, and cranial CT/MRI confirmed leptomeningeal capillary malformations with calcifications in the left occipital lobe (no cerebral atrophy). The patient was diagnosed with SWS type III based on clinical and imaging findings, and his symptoms were well-controlled with symptomatic treatment. This case highlights the importance of imaging modalities in diagnosing SWS type III in pediatric patients with non-specific neurological symptoms, and emphasizes the favorable prognosis of early-detected cases with short disease courses.

## 1 Introduction

Sturge-Weber syndrome (SWS) is a rare, sporadic neurocutaneous disorder with an incidence of ∼1 in 50,000 individuals (1). Owing to its rarity and heterogeneous clinical manifestations, it has long attracted the attention of clinicians (2).Classified into three subtypes according to the involvement of skin, eyes, and central nervous system (CNS), SWS type I is the most common variant, characterized by the classic triad of facial port-wine stain (typically in the V1/V2 distribution of the trigeminal nerve), ocular abnormalities (e.g., glaucoma, choroidal hemangioma), and leptomeningeal angiomatosis with CNS calcifications and atrophy. SWS type II is defined by the coexistence of facial port-wine stain and ocular involvement, but without CNS lesions. By contrast, SWS type III is the rarest variant, defined by isolated neurological involvement (e.g., epilepsy, headache) without typical facial port-wine stain or ocular abnormalities (3). Neurological complications are common in approximately 70%–80% of SWS patients, including epilepsy, hemiplegia, stroke-like episodes, intellectual disability, and behavioral problems. According to existing studies, epilepsy and headaches are the most prevalent neurological manifestations in SWS type III pediatric patients. It may also present with other signs of SWS (2, 4, 5). Due to its non-specific clinical presentation, SWS type III is often underdiagnosed in pediatric populations, and imaging plays a pivotal role in confirmation. Here, we report a case of SWS type III in a 5-year-old boy to illustrate the clinical and imaging features of this rare subtype, and discuss its diagnosis and management in line with current literature.

## 2 Case presentation

A 5-year-old male was admitted to our hospital with a 1-year history of recurrent headaches and convulsions. The patient first developed headaches one year prior to admission, which resolved spontaneously. Several days later, he presented with convulsions and vomiting, with 6–7 convulsive episodes occurring within a 24-h period. His symptoms were relieved after symptomatic treatment at another hospital. Following discharge, he was administered oral valproic acid for approximately two months, but the medication was discontinued voluntarily by his family. Intermittent headaches persisted thereafter, and the frequency of headache episodes increased over the past two months. The patient was subsequently admitted to our hospital for further management, and experienced clustered epileptic seizures within a 24-h window during the current hospitalization. No relevant family history of neurological disorders was reported.

### 2.1 Physical examination

No facial erythema, port-wine stain, or postural abnormalities were observed. Ocular examination was unremarkable (no glaucoma or choroidal hemangioma).

### 2.2 Laboratory tests

Blood ammonia was 29.4 μmol/L (reference: 10–27 μmol/L), pyruvate 18 μmol/L (reference: 20–100 μmol/L); other routine blood and biochemical tests were normal.

### 2.3 Electroencephalogram (EEG)

Video EEG demonstrated a significant increase in slow wave activity across all leads, with no focal epileptiform discharges.

### 2.4 Imaging findings

**Figure 1:**
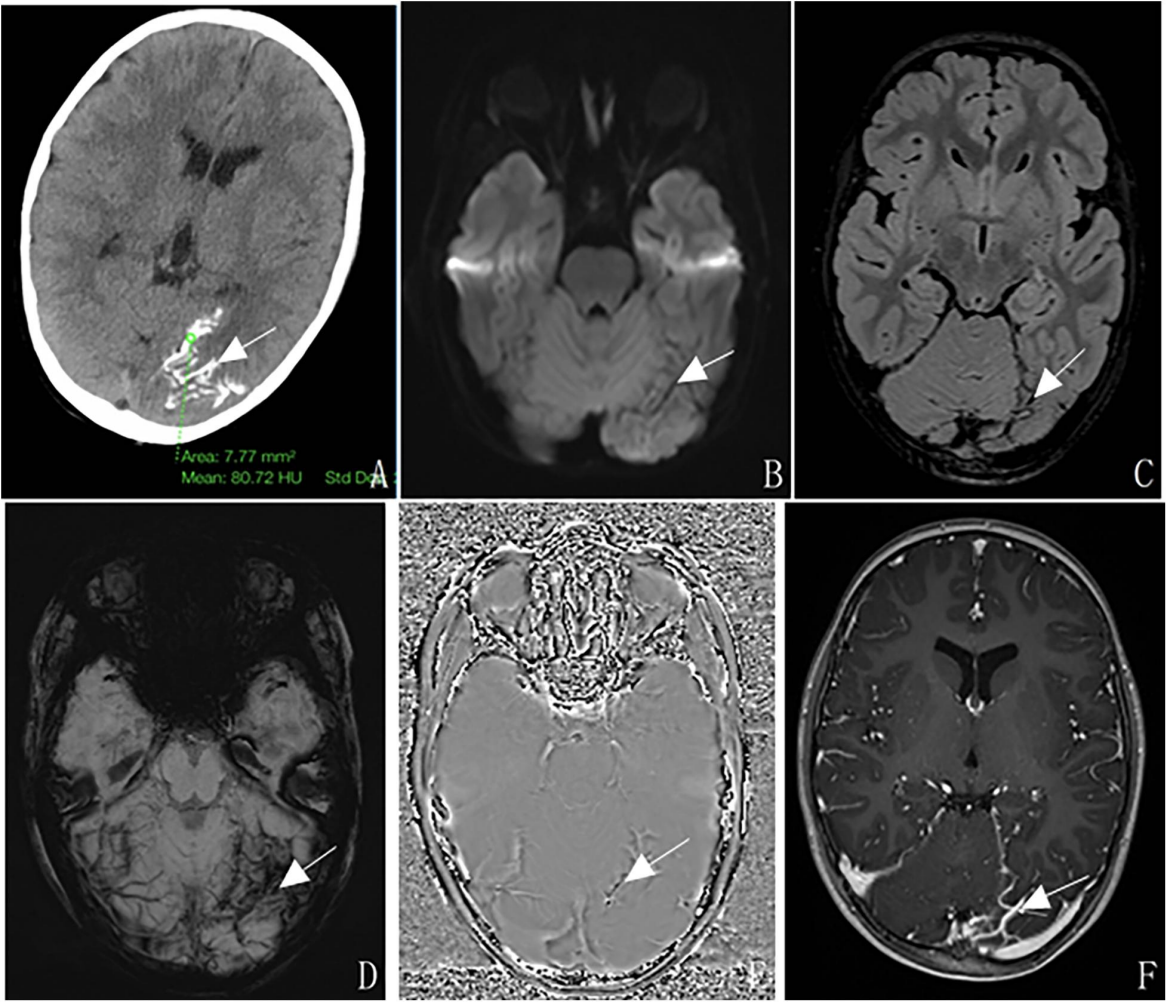
The patient is a 5-year-old male with Sturge-Weber syndrome type III. CT shows multiple tortuous vascular-like calcifications along the sulcus course of the left occipital lobe **(A, arrow)**. MRI shows multiple tortuous strip-shaped low signals on DWI **(B, arrow)** and FLAIR **(C, arrow)**; SWI images **(D, arrow)** and phase images **(E, arrow)** show multiple strip-shaped low signals in the left occipital lobe; After enhancement, there was a significant increase in tortuosity of the left occipital leptomeningeal vessels with enhancement **(F, arrow)**.

Cranial CT (axial view): Multiple tortuous vascular-like calcifications along the sulcal course of the left and occipital lobe, no cerebral atrophy, and mild asymmetry of bilateral occipital lobes (Figure 1A). MRI: FLAIR/DWI sequences showed multiple tortuous strip-shaped hypointensities in the left occipital lobe (Figures 1B,C); T1WI/T2WI sequences were unremarkable; SWI confirmed multiple calcifications (Figures 1D,E); contrast-enhanced MRI revealed marked tortuosity and enhancement of left occipital pial meningeal vessels (arrows), with tortuous thickened venous shadows associated with the straight sinus, confluence of sinuses, left sigmoid sinus; no choroid plexus enlargement (Figure 1F).

## 3 Diagnosis and management

Based on the clinical presentation (recurrent headache/convulsions, no cutaneous/ocular involvement) and typical imaging features of leptomeningeal capillary malformation with calcifications, a diagnosis of SWS type III was made. The patient received symptomatic treatment (analgesics and antiepileptic medication). The most commonly used medications for acute headaches include nonsteroidal anti-inflammatory drugs (NSAIDs) such as ibuprofen and acetaminophen, as well as triptans such as sumatriptan (5). Currently, there are no established management guidelines for epilepsy treatment in Sturge-Weber syndrome (SWS). Studies have shown that levetiracetam, oxcarbazepine, and phenobarbital are the most frequently used antiepileptic drugs (AEDs). Lacosamide and topiramate are less commonly utilized and are typically administered as adjunctive antiepileptic agents (6). In this case, the patient was prescribed ibuprofen granules at a dose of 5 mg/kg per dose, taken three times daily during hospitalization administered for headache relief, as well as oral levetiracetam, initiated at a dose of 10 mg/kg/day and titrated to 20 mg/kg/day after 1 week). Additionally, the patient was advised to avoid strenuous exercise to reduce headache triggers. His symptoms resolved with no recurrent seizures during hospitalization. At discharge, his condition was stable, and long-term follow-up was scheduled to monitor the condition.

## 4 Discussion

SWS type III is distinguished by exclusive neurological involvement, and headache is a common initial symptom in pediatric patients. The seizures are predominantly generalized in type (7–9). Consequently, the clinical presentation of this disease is atypical, rendering early diagnosis challenging. Electroencephalography (EEG) can facilitate the diagnosis of SWS; screening for cerebral involvement in SWS using EEG prior to the onset of clinical symptoms may enable early identification. EEG often shows diffuse slow wave abnormalities, which aligns with our patient's findings (10, 11). While biopsy is the gold standard for diagnosis, its invasiveness limits use in children; thus, neuroimaging (CT/MRI) is the primary diagnostic tool (8). A retrospective study of 10 pediatric SWS type III cases at Boston Children's Hospital reported pial vascular malformations (with/without deep venous abnormalities) as the main imaging feature, with calcifications and atrophy present in subsets of patients (9). Our patient had calcifications but no atrophy, likely due to his short disease course—consistent with reports that early-stage SWS type III may lack structural brain damage (12).

## 5 Differential diagnosis

Given the non-specific clinical manifestations of SWS type III (recurrent headache and epilepsy) and imaging features (leptomeningeal calcifications and vascular malformations), several pediatric neurological and neurovascular disorders need to be excluded:

1) Intracranial infections (e.g., viral encephalitis, tuberculous meningitis): These disorders may present with headache and seizures, and imaging may show leptomeningeal enhancement. However, they are usually accompanied by fever, elevated inflammatory markers, and CSF abnormalities, which were absent in our patient. 2) Cerebral Cavernous Malformation (CCM): Multiple CCMs (especially familial cases) cause recurrent hemorrhage, seizures, and focal deficits; MRI shows “popcorn” lesions with hemosiderin deposition: CCMs are discrete vascular nodules without diffuse leptomeningeal enhancement or progressive atrophy (13). 3) Neurofibromatosis Type 1 (NF1) with Focal Leptomeningeal Abnormalities: NF1 may present with focal areas of leptomeningeal thickening or enhancement, mimicking SWS III. However, these are typically non-progressive and lack the tram-track calcifications characteristic of SWS. Café-au-lait macules, cutaneous neurofibromas, Lisch nodules, and optic pathway gliomas. NF1-related leptomeningeal changes are not angiomatous and do not lead to the progressive cortical atrophy seen in SWS (14).

## 6 Conclusion

This case illustrates the typical clinical and multimodal imaging features of SWS type III in a pediatric patient, highlighting the pivotal role of neuroimaging in diagnosing this rare subtype without cutaneous manifestations. CT is valuable for detecting calcifications, while MRI (especially SWI and contrast-enhanced sequences) provides critical information about leptomeningeal vascular malformations. By optimizing imaging protocols and recognizing these characteristic findings, radiologists can contribute to the early diagnosis and management of SWS type III, ultimately improving patient outcomes. Long-term follow-up is essential to monitor disease progression and adjust clinical management accordingly.

## Data Availability

The original contributions presented in the study are included in the article/Supplementary Material, further inquiries can be directed to the corresponding author.
